# Is nanomaterial- and vancomycin-loaded polymer coating effective at preventing methicillin-resistant *Staphylococcus aureus* growth on titanium disks? An in vitro study

**DOI:** 10.1007/s00264-023-05757-2

**Published:** 2023-03-28

**Authors:** Konstantinos Tsikopoulos, Gabriele Meroni, Panagiotis Kaloudis, Eleni Pavlidou, Christoforos Gravalidis, Ioannis Tsikopoulos, Lorenzo Drago, Carlo Luca Romano, Paraskevi Papaioannidou

**Affiliations:** 1 1st Department of Pharmacology, School of Medicine, Faculty of Health Sciences, Aristotle University of Thessaloniki (AUTh), Thessaloniki, Greece 54124; 2 One Health Unit, Department of Biomedical Surgical and Dental Sciences, School of Medicine, Università degli Studi di Milano, Milan, Italy; 3 Condensed Matter and Materials Section, Department of Physics, Faculty of Exact Sciences, Aristotle University of Thessaloniki (AUTh), Thessaloniki, Greece 54124; 4 Laboratory of Clinical Microbiology and Microbiome, Department of Biomedical Sciences for Health. School of Medicine, University of Milan, Milan, Italy; 5 Studio Medico Associato Cecca-Romanò, Corso Venezia 2, Milan, Italy

**Keywords:** Methicillin-resistant *Staphylococcus aureus*, Al_2_O_3_ nanowires, TiO_2_ nanoparticles, Vancomycin

## Abstract

**Purpose:**

Periprosthetic joint infections induced by methicillin-resistant *Staphylococcus aureus* (MRSA) pose a major socioeconomic burden. Given the fact that MRSA carriers are at high risk for developing periprosthetic infections regardless of the administration of eradication treatment pre-operatively, the need for developing new prevention modalities is high.

**Methods:**

The antibacterial and antibiofilm properties of vancomycin, Al_2_O_3_ nanowires, and TiO_2_ nanoparticles were evaluated in vitro using MIC and MBIC assays. MRSA biofilms were grown on titanium disks simulating orthopedic implants, and the infection prevention potential of vancomycin-, Al_2_O_3_ nanowire-, and TiO_2_ nanoparticle-supplemented Resomer® coating was evaluated against biofilm controls using the XTT reduction proliferation assay.

**Results:**

Among the tested modalities, high- and low-dose vancomycin-loaded Resomer® coating yielded the most satisfactory metalwork protection against MRSA (median absorbance was 0.1705; [IQR = 0.1745] vs control absorbance 0.42 [IQR = 0.07]; *p* = 0.016; biofilm reduction was 100%; and 0.209 [IQR = 0.1295] vs control 0.42 [IQR = 0.07]; *p* < 0.001; biofilm reduction was 84%, respectively). On the other hand, polymer coating alone did not provide clinically meaningful biofilm growth prevention (median absorbance was 0.2585 [IQR = 0.1235] vs control 0.395 [IQR = 0.218]; *p* < 0.001; biofilm reduction was 62%).

**Conclusions:**

We advocate that apart from the well-established preventative measures for MRSA carriers, loading implants with bioresorbable Resomer® vancomycin-supplemented coating may decrease the incidence of early post-op surgical site infections with titanium implants. Of note, the payoff between localized toxicity and antibiofilm efficacy should be considered when loading polymers with highly concentrated antimicrobial agents.

**Supplementary Information:**

The online version contains supplementary material available at 10.1007/s00264-023-05757-2.

## Introduction

Methicillin-resistant *Staphylococcus aureus (*MRSA) infection correlates with significant morbidity, mortality, length of stay, and cost burden [1]. From a pathophysiological perspective, high-level resistance to β-lactam antibiotics in (MRSA) is due to the expression of penicillin-binding protein 2a (PBP2a), with *mecA* and *mecC* genes being implicated in the pathogenesis of this condition [2]. It is worthy of note that community acquired MRSA is different from healthcare-acquired MRSA in nature as they tend to affect a different group of patients, and therefore treatment requirements differ accordingly [3]. Orthopedic literature has focused on preventing surgical site infection in this group of patients, given that MRSA carriers run a two to nine times higher risk of developing this complication than the normal population [4].

While many infection prevention methods have been developed over the last years in the orthopaedic setting, the best strategy has yet to be determined. Coatings of internal fixation implants as a protection measure hold promise, with various technologies available to include passive surface modification, organic or inorganic or synthetic active surface finishing, and biodegradable or non-biodegradable local carriers [5]. A recent meta-analysis of animal literature showed that combining active surface finishing (i.e., coating with pharmacologically active bactericidal compounds) and passive coating (i.e., surface modifications without using any pharmacologically active agents) results in the best infection prevention outcomes against *S. aureus* when titanium implants are present [6].

In terms of the constituents of surface finishing, consideration of nanomaterials appears to be advantageous over conventional active coating for multiple reasons, including, but not limited to, better control of drug release and acting at a subcellular level [7]. Therefore, in the present study, we sought to investigate the antibiofilm potential of vancomycin- and nanomaterial-loaded biodegradable coating using an in vitro infection model with titanium disks simulating orthopaedic implants.

## Methods

### Setting the experiment

A clinical MRSA strain isolated from an infected joint replacement at Hippokration Hospital (Thessaloniki, Greece) was used in all experiments. We studied the biofilm growth capacity of *S. aureus* by using scanning electron microscopy (SEM) at the Department of Physics, Faculty of Exact Sciences, Aristotle University of Thessaloniki (Thessaloniki, Greece). Most of the nanomaterial experiments were conducted at the 1st Department of Pharmacology of the Medical School of Aristotle University of Thessaloniki (Thessaloniki, Greece). In addition, the biofilm-related experiments were conducted at the Hippokration Hospital, Thessaloniki, Greece, and Institutional Board Approval was obtained (IRB 22049/6-5-2022).

### Nanomaterials and antimicrobial agents

The following antibacterial substances were used: vancomycin (Fresenius, Kabi, Bad Homburg, Germany), Al_2_O_3_ nanowires (diameter × L 2–6 nm × 200–400 nm Sigma-Aldrich in powder form), and TiO_2_ nanoparticles (Nanografi, Turkey).

### Biomaterials

Sandblasted medical-grade titanium alloy disks (Ti6Al4V, diameter 4 mm, height 2 mm) were implemented to enable the simulation of orthopaedic implants utilized in a cementless mode of fixation. Moreover, the biomaterials’ roughness was quantified by SEM (FESEM-JSM-7610 Fplus Thermal, Analytical FE SEM).

### Minimum inhibitory concentration (MIC) determination

The clinical and laboratory standard guidelines were followed to assess the tested agents minimum inhibitory concentration (MIC) [8]. In brief, some isolated colonies of *S. aureus* were recovered from mannitol salt agar (bioMérieux, Marcy I***'***Etoile, France) and subsequently resuspended in tryptic soy broth (NutriSelect® Plus, Sigma-Aldrich, Schnelldorf, Germany) to an optical density of 0.5 McFarland (ca 1.5 × 10^8^ colony forming [CFU] units per mL). This suspension was then dispersed in a 96-well microplate containing serial twofold dilutions of the testing molecules to a final concentration of 10^5^ CFU/mL. MIC values were visually assessed after 48 h. The following antibacterial agents were assessed against the planktonic form of *S. aureus*: (1) vancomycin (Vianex, Greece); (3) Al_2_O_3_ nanowires (diameter × L 2–6 nm × 200–400 nm; Sigma); (3) TiO_2_ nanoparticles (Nanografi, Turkey); daptomycin (Accord Healthcare SLU); Fucidic Acid (LEO Pharmaceutical); linezolid (Pfizer, Hellas A.E.). The above compounds were diluted in a twofold fashion with NaCL 0.9%.

### Coating of biomaterials

The rationale behind the contents of our coating gel was to avoid toxicity secondary to excessive use of DMSO and bactericidal effects stemming from alcohol. Therefore, 20 mL of coating gel was produced as follows: 10 mL of 90% alcohol was added to the Resomer® powder (Poly(D,L-lactide) (PDLLA) acid terminated, Mw 18,000–24,000 g/mol), nanoparticles resuspended in 2 mL of DMSO, and the remaining 8 mL included water for injection.

To achieve even coating on the surface of titanium disks (Supplemental file 1), we implemented a novel technique that consisted of air-brush spraying to cover the whole of the biomaterials with PDLLA alone or supplemented with antibacterial agents. More specifically, we considered a nozzle-to-substrate distance of 20 cm, an appropriate spraying pressure of 1 bar, and a continuous spraying time of 60 s. Of note, the air brush was placed completely vertically relative to the disks to enable the formation of a spraying cone with a radius of ~ 60 mm, and after completing the procedure, films were dried in the air with no further thermal processing. Notably, disks were aseptically weighed before and after application for experiment reproducibility reasons, and a median increase of 2.2 mg (interquartile range = 0.8) was demonstrated.

### Biofilm production and minimum biofilm inhibition concentration (MBIC) determination

Before conducting any biofilm experiments, we confirmed the ability of our MRSA clinical isolate to form mature biofilm by staining their matrix with safranin (200 µL of 0.1% safranin for 5 min), rinsing and measuring spectrophotometric absorbance at 492 nm (Epoch™ BioTek, Winooski, VT, USA). The extent of biofilm formation was measured with SEM (FESEM-JSM-7610 Fplus Thermal, Analytical FE SEM). The samples were mounted on bronze substrates with adhesive double-sided carbon tape, and were then subjected to coating with carbon featuring an average thickness of 200 Å using a vacuum evaporator JEOL 4X. For this study, the minimum biofilm inhibitory concentration (MBIC) was determined using a similar technique to that used for planktonic bacteria [9]. The following coating combinations were considered: (1) 4xMIC and 16xMIC of vancomycin (Vianex, Greece); (2) 4xMIC and 16xMIC of Al_2_O_3_ nanowires (diameter × L 2–6 nm × 200–400 nm; Sigma); and (3) 4xMIC and 16xMIC of TiO_2_ nanoparticles (Nanografi, Turkey).

To assess the metabolic activity of biofilm or planktonic MRSA cells, we implemented the XTT metabolic-reduction assay (ThermoFisher Scientific). Well plates were incubated for 48 h and subsequently subjected to centrifugation at 4000 rpm for 30 min. The supernatant was then discarded and substituted with XTT solution. After that, a solution of PBS containing 0.25 mg/mL XTT and 40 µg/mL coenzyme Q_0_ was incubated at 37 °C for 1 h. Then, 100 µL of this solution was withdrawn and transferred to a new well plate for spectrophotometrical reading. An automated plate reader (Epoch^TM^BioTek, Winooski, VT, USA) measured absorbance at 450 nm with a reference wavelength of 690 nm. Percent metabolic activity was calculated with the following equation: (1 − *X*/*C*) × 100, where *X* is the OD of agent-containing wells and *C* is the OD of control wells with fungi only.

### Statistical analysis

Statistical software SPSS 29.0 (SPSS, Chicago, IL, USA) was used for the data analyses. A non-normal distribution was revealed following normality assessment, and Mann–Whitney and Kruskal–Wallis tests were implemented for two and multiple-group comparisons, in that order.

The sample size was determined concerning published guidelines for in vitro research [10], and the calculation depended on the previously described minimal clinically meaningful reduction rate of 80% for biofilm growth [11]. Therefore, the required statistical power was set at 0.8, with a and b errors being 5% and 20%, respectively. Ultimately, we found that at least 8 disks per intervention group were needed to reach sufficient statistical power. For the statistical analyses, a *p*-value < 0.5 demonstrated statistical significance and GraphPad Prism 9 (GraphPad Software, Inc, La Jolla, CA, USA) software was utilized for graph creation.

## Results

### Minimum inhibitory concentration (MIC) and minimum biofilm inhibition concentration (MBIC) assessment

The MIC values of daptomycin, linezolid, and fucidic acid were found to be 0.5, 2, and 16 µg/mL respectively. Among Al_2_O_3_ nanowires, TiO_2_ nanoparticles, and vancomycin, the latter was found to be the most effective agent at preventing MRSA biofilm growth (Table 1).

**Table 1:** Minimal inhibitory concentrations of the major antibacterial agents

Antibacterial agent	MIC (µg/mL)	MBIC (µg/mL)
Al_2_O_3_ nanowires TiO_2_ nanoparticles Vancomycin	1024 1024 0.5	4096 8192 4

*MBIC*, minimum biofilm inhibitory concentration; *MIC*, minimum inhibitory concentration

### *Surface topography of titanium disks*

**Fig. 1:**
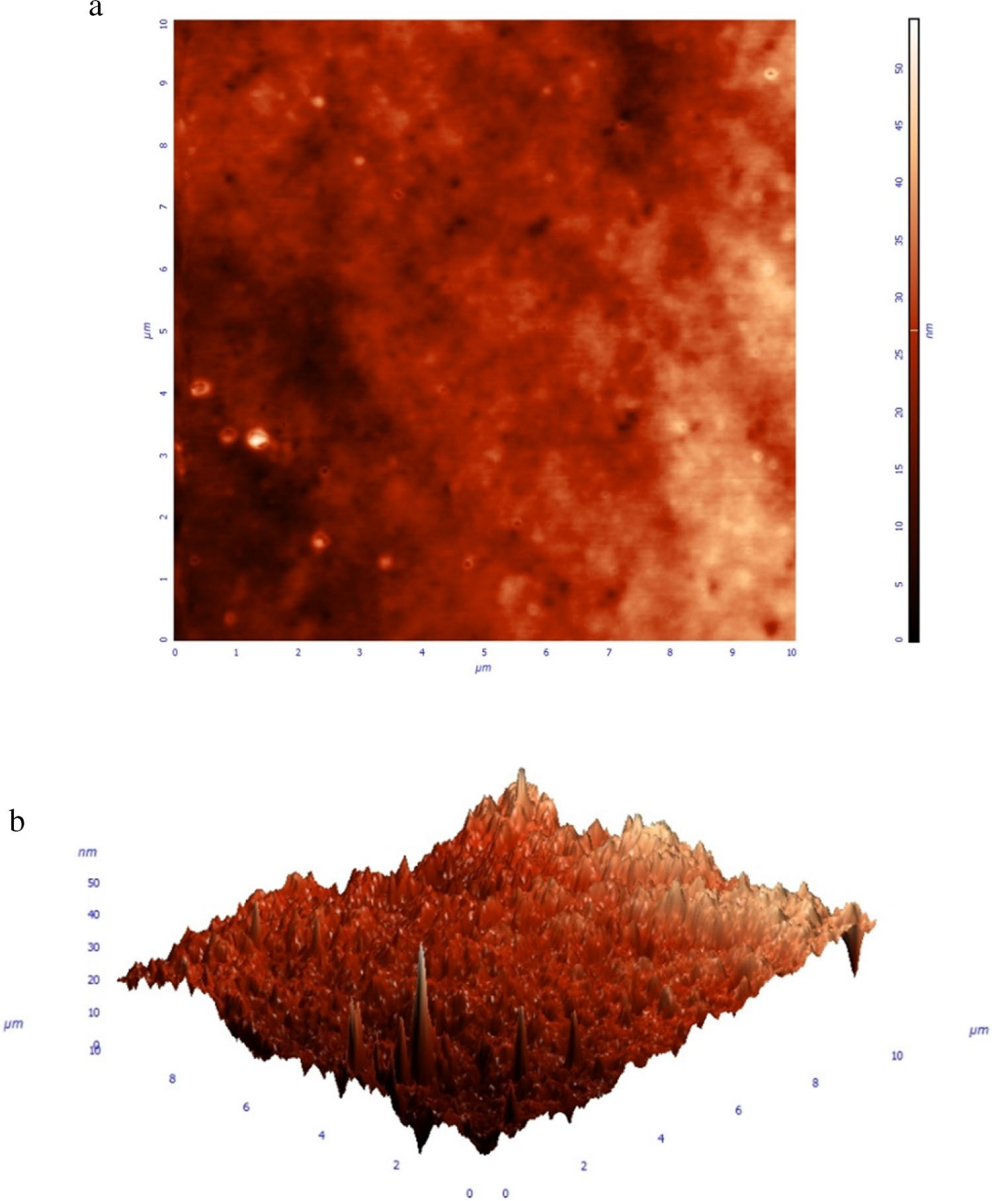
**a** Axial view demonstrating titanium disk roughness assessment using atomic force microscopy. **b** 3D view depicting titanium disk roughness by means of atomic force microscopy

AFM (AFM Solver, NT-MDT) was used to investigate the surface topography and roughness of the spray coated samples in the tapping mode. For the experiments, image acquisition was performed using rectangular silicon cantilevers with a 6 nm nominal tip curvature and a force constant of 5.1 nm^−1^. Physical discontinuities of titanium disks were quantified prior to coating experiments, and roughness was found to be 5.65 Ra with a peak-to-valley height measuring 54.12 nm (Fig. 1a, b).

### Mature biofilm production confirmation

**Fig. 2:**
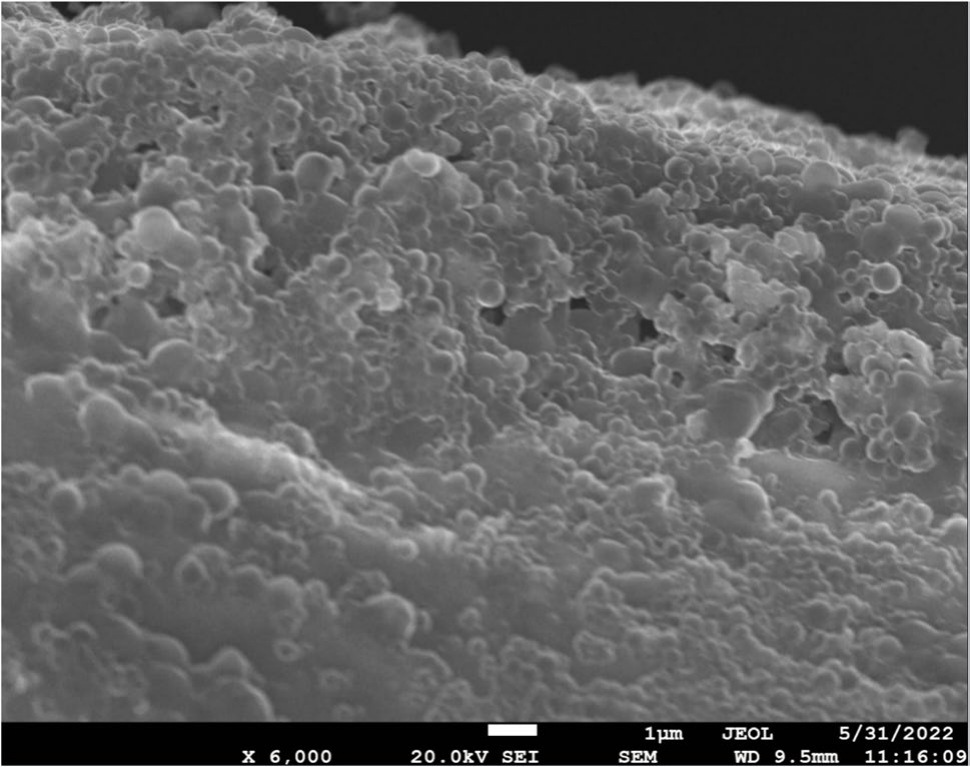
Biofilm formation on a titanium disk as depicted with SEM

After determining the inhibitory concentration, we used SEM to establish that the MRSA strain could create a mature biofilm. After this process, it was demonstrated that the tested strain was a strong biofilm producer (Fig. 2).

### Impact of Resomer® coating on MRSA biofilm growth

**Fig. 3:**
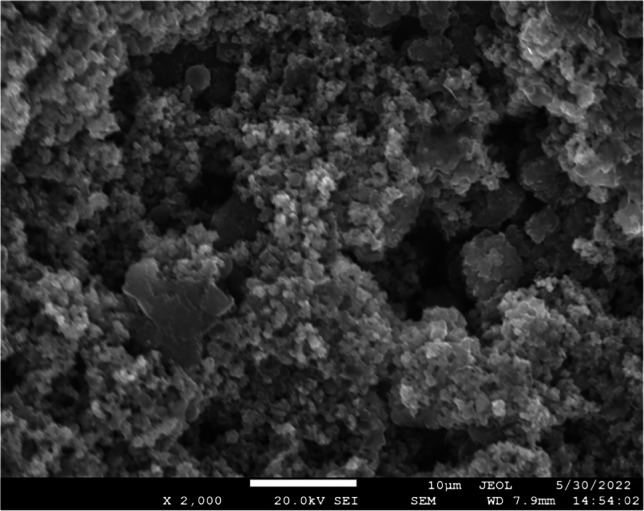
SEM image depicting biofilms in addition to PDLLA coating on a titanium disk

High-dose (i.e., 16xMIC) and low-dose (i.e., 4xMIC) concentrations were tested to assess the antibiofilm potential of Al_2_O_3_ nanowires, TiO_2_ nanoparticles, and vancomycin (Fig. 3).

Among the tested coating options, both high- and low-dose vancomycin titanium coatings were proven effective at preventing MRSA infection in vitro, as they both exceeded the minimal clinically important difference of 80% in biofilm reduction relative to biofilm control (Table 2).

**Table 2:** Αnti-biofilm potential of various coating modalities.

Treatment group	Median absorbance (IQR)		*p*-value	Biofilm reduction
	Intervention group	Biofilm control		
High-dose Al_2_O_3_-loaded coating	0.2255 (0.052)	0.395 (0.218)	0.002	77%
Low-dose Al_2_O_3_- loaded coating	0.2375 (0.233)	0.395 (0.218)	0.023	72%
High-dose TiO_2_-loaded coating	0.22 (0.09)	0.42 (0.07)	< 0.001	80%
Low-dose TiO_2_-loaded coating	0.2405 (0.169)	0.42 (0.07)	0.005	72%
High-dose vancomycin-loaded coating	0.1705 (0.175)	0.42 (0.07)	0.016	100%
Low-dose vancomycin-loaded coating	0.209 (0.13)	0.42 (0.07)	< 0.001	84%
Resomer® coating alone	0.2585 (0.124)	0.395 (0.218)	< 0.001	62%

**Fig. 4:**
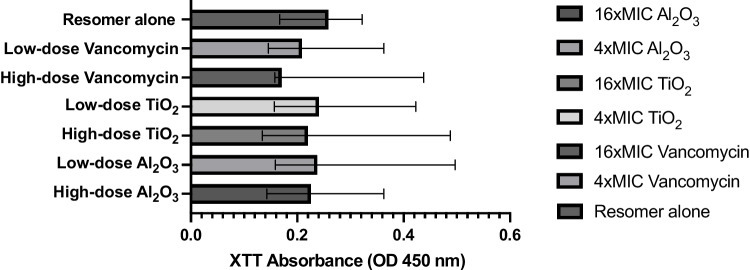
Graphical presentation of the effects of Resomer.® coating on MRSA biofilm development using XTT reduction assay. MIC, minimum inhibitory concentration; OD, optical density; XTT, (2,3-bis(2-methoxy-4-nitro-5-sulfophenyl)-5-carboxanilide-2H-tetrazolium)

Between-group comparisons showed no statistically significant differences (*p* = 0.749). To be more specific, no statistically significant differences were detected when high-dose vancomycin-loaded Resomer® was compared against PDLLA coating alone (*p* = 0.345) (Fig. 4). Likewise, there was no statistically significant difference between high- and low-dose Al_2_O_3_-, TiO_2_-, and vancomycin-loaded coatings (*p*-values were 0.713, 0.431, and 0.713, respectively).

## Discussion

The management of MRSA device-associated infections represents a major challenge for orthopaedic surgeons. Unfortunately, despite the recent advances in preventing the above complication, complete device protection cannot be guaranteed with the existing infection-preventative measures. Therefore, in the present in vitro study, we sought to assess the antibiofilm potential of novel modalities, including vancomycin-, Al_2_O_3_ nanowire-, and TiO_2_ nanoparticle-supplemented Resomer® coating against MRSA growth on titanium disks simulating orthopaedic implants. We showed that loading implants with low- or high-dose bioresorbable PDLLA vancomycin-supplemented coating can significantly diminish bacterial load enclosed in the biofilm matrix, which could be potentially useful in decreasing the incidence of early post-op surgical site infections. In other words, successful antiadhesion and bactericidal performance were exhibited, thus rendering MRSA bacteria unable to settle on the implant’s surface and win the “race for the surface.” However, this finding should be interpreted with caution, given the preliminary in vitro nature of the current investigation.

### Requirements for setting up an orthopedic experiment

Given the experimental nature of nanocoating components against MRSA, a basic in vitro study design was carefully selected to address this clinically important orthopaedic research question. From an administrative point of view, Institutional Review Board Approval was required from the local General Hospital prior to conducting microbiological experiments. On top of that, up-front investment in infrastructure and consumables was needed to ensure smooth lab working conditions. From a scientific standpoint, familiarity with basic lab skills in addition to close collaboration with well-established infection-dedicated labs/Institutions for remote supervision of the planning and execution of experiments was achieved in advance of this investigation.

### Why coating implants to prevent MRSA implant infections?

Taking into account that the average cost of managing MRSA infections varies between $3.2 and $4.2 billion [12] and considering that the annual death burden of MRSA in the USA has increased to 11,000 [13], the need for adopting new more effective prevention modalities in the orthopaedic settings is underlined. In optimizing the potential of infection prophylaxis, separating patients from high- and low-risk ones depending on whether they are MRSA carriers is supported by the existing literature [3]. For MRSA carriers, timely antibiotic administration featuring vancomycin 15 mg/kg (or teicoplanin) 60 min before the skin incision [3], optimization of comorbidities and nutrition, meticulous soft tissue handling in the operating theatre in addition to utilizing isolation suits, reducing the circulating theatre personnel, and side room management post-operatively are necessary. Nevertheless, the above measures are sometimes insufficient; therefore, more research is needed to identify adjuvant methods to boost their efficacy.

### To decolonize or not decolonize MRSA-positive patients pre-operatively?

First of all, it is undeniable that MRSA-colonized patients are at a higher risk for developing a surgical site infection than other patients undergoing elective surgery, particularly when it comes to lower limb arthroplasty [14]. In that scenario, should a post-operative infection occur, it would be more likely to be secondary to MRSA [15].

However, it is underlined that there is a lot of controversy on the need for pre-operative decolonization of MRSA-positive patients, which is reflected in the fact that this prophylactic treatment is not widely performed in the USA as opposed to the UK. To illustrate, despite the common belief that eradication of nasal carriage reduces the incidence of MRSA infection, recent randomized evidence has failed to prove any significant difference between commonly used protocols (i.e., mupirocin and chlorhexidine body washes) relative to untreated controls [16]. On top of that, it is highlighted that the use of vancomycin in non-MRSA carriers may result in increased post-surgical infections [17]. Therefore, caution is advised when administering this particular antibiotic in the above group of patients.

### Biomaterial selection

In the current study, we opted to investigate Ti6AL-4 V because medical-grade titanium and its alloys have been the most extensively used biomaterials in orthopaedic clinical practice since 1950 [18]. It is worth mentioning that coating is only applicable in biological implant fixation, for which titanium is one of the most appropriate options due to its excellent biocompatibility and young modulus of elasticity [18, 19]. In terms of the impact of coating on titanium implant osseointegration, it should be noted that an appropriate biomaterial roughness with the right mechanical properties could improve results relative to an uncoated titanium implant [20]; however, this research field requires further in-depth investigation. At this point, we wish to draw the reader’s attention to the fact that the in vitro nature of the present study in conjunction with the small dimensions of the above disks allowed for multiple comparisons between intervention groups and sufficient study power.

### Nanomaterials as coating agents

Due to the long-term sequela of excessive use of vancomycin that inevitably results in resistance development [21], we sought to utilize nanomaterials as active coating components. Theoretically, nanowires (i.e., structures with a thickness or diameter constrained to tens of nanometers or less and an unconstrained length) are ideal coating materials due to their size, high surface area, surface chemistry, and satisfactory adherence to surfaces. Considering that Al_2_O_3_ nanowires have not been previously investigated at a very basic in vitro orthopedic research setting and considering that they appear less toxic than Al_2_O_3_ nanoparticles [22], we decided to assess the efficacy of the above nanomaterial against MRSA biofilms.

TiO_2_ was chosen among the commercially available nanoparticles because of its outstanding biocompatibility, resistance to corrosion, affordability, non-toxicity, strong self-cleaning capability, and potential for antibacterial activity against *Staphylococcus* [23, 24]. Despite its promising antibacterial efficacy as a coating agent demonstrated in earlier literature [24], the present study showed that TiO_2_ coating could not yield sufficient metalwork protection against MRSA biofilm growth.

In terms of passive coating constituents, the rationale behind selecting Resomer® was that degradation of a given polymer takes place over the course of several months; however, the longevity of this method relies upon the implantation site and the thickness of the polymer film [25]. On top of that, in vitro evidence has suggested that PDLLA coating does not exert a negative impact on the T-cell-regulated local immune system [26].

### Study limitations and implications for future research

We recognize that the present study presents a few limitations. First, the in vitro nature of this article does not allow for direct extrapolations to complex human biology. In other words, caution should be exercised when interpreting the results of the present investigation and we advise that further research should be conducted before adopting our suggested coating techniques in orthopaedic clinical practice. Second, we note that localized coating toxicity was not investigated in the current study as this was beyond the scope of our work. However, we claim that future research should mainly focus on the safety profile of self-dissolving vancomycin coating to provide clinicians with a more comprehensive profile of this modality. Third, animal model studies should be conducted to verify the efficacy of the proposed combined coating and assess whether or not implant coating with vancomycin exerts a negative impact on implant osseointegration and osteoblasts [28]. Fourth, we contend that while early post-operative implant infections are prevented because they are less frequent than late infections, creating a more durable coating would enable the continuous release of active antibacterial agents throughout time. On top of that, recent evidence has suggested that applying a multilayer coating on titanium implants may be advantageous over conventional coating [27]. Last but not least, we note that only one clinical MRSA strain was considered in the current investigation, and results were not compared against a reference control (i.e., the standard MRSA strain ATCC 43,300). In addition, to achieve an in-depth investigation of the comparators in this study, no further antibacterial substances were considered.

## Conclusions

Vancomycin-loaded Resomer® coating effectively prevented biofilm MRSA growth on titanium disks in vitro in the tested concentrations. In relating this finding to orthopaedic clinical practice, the application of the coating on titanium implants could be considered as a further preventative measure against MRSA infection development in orthopaedics.

## Supplementary Information

Below is the link to the electronic supplementary material.

Supplementary file1 (PDF 38 KB)

## Data Availability

Raw data are available on request.
