# Supplementary figures and images for: Is nanomaterial- and vancomycin-loaded polymer coating effective at preventing methicillin-resistant Staphylococcus aureus growth on titanium disks? An in vitro study
Source: Int Orthop. 2023 Mar 28;47(6):1415–22. doi: 10.1007/s00264-023-05757-2 (PMC10199848; doi:10.1007/s00264-023-05757-2)

**Supplemental file 1.** Comparison between a plasma-sprayed coated and an uncoated titanium disk

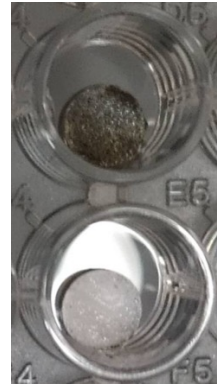

Supplement: Supplementary file 1 — Supplementary file1 (PDF 38 KB) [file 264_2023_5757_MOESM1_ESM.pdf]
